# Glial pathology networks reveal early olfactory vulnerability in *post mortem* human Alzheimer's disease

**DOI:** 10.1002/alz.71322

**Published:** 2026-04-06

**Authors:** Da Hae Jung, Eunji Park, Hyeon Chang Ju, Cheil Moon, Ali Jahanshahi

**Affiliations:** ^1^ Department of Brain Sciences Graduate School, Daegu Gyeongbuk Institute of Science and Technology (DGIST) Daegu Republic of Korea; ^2^ Convergence Research Advanced Centre for Olfaction, Daegu Gyeongbuk Institute of Science and Technology (DGIST) Daegu Republic of Korea; ^3^ Department of Neurosurgery Maastricht University Medical Center Maastricht the Netherlands

**Keywords:** Alzheimer's disease, astrocytes, microglia, olfactory bulb, olfactory cortex, protein aggregation

## Abstract

**INTRODUCTION:**

The olfactory system is an early target in Alzheimer's disease (AD), yet regional glial pathology interactions remain poorly defined. We examined how glial activation and pathological burden differ between the olfactory cortex (OC) and olfactory bulb (OB) across disease stages.

**METHODS:**

*Post mortem* OC and OB samples from cognitively normal (CN), mild cognitive impairment, and AD cases were analyzed using immunohistochemistry and immunofluorescence for amyloid beta (Aβ), phosphorylated tau (pTau), Iba1 (microglia), GFAP (astrocyte), and apolipoprotein E (apoE).

**RESULTS:**

Both regions showed stage‐dependent increases in Aβ and pTau, with regionally distinct glial responses. ApoE signal varied with clinical stage rather than genotype. Co‐expression analyses revealed astrocyte‐linked networks in the OC and microglia‐linked relationships in the OB.

**DISCUSSION:**

Findings demonstrate spatially heterogenous glial pathology architectures in the human olfactory system, supporting its role as an early and regionally diverse site of AD vulnerability.

## Highlights

- Progressive Aβ and pTau burden observed in both OC and OB across AD stages.
- Glial morphology exhibits stage‐related changes across regions.
- Co‐expression patterns differ between OC and OB.
- Findings support early and region‐specific vulnerability of the olfactory system.

## 1 BACKGROUND

Olfactory dysfunction is a frequent and often prodromal symptom of Alzheimer's disease (AD), emerging before overt cognitive decline and offering a window into early pathophysiological changes.^1^, ^2^, ^3^ Neuropathological studies consistently show that olfactory‐related regions – including the olfactory cortex (OC) and olfactory bulb (OB) – are among the first to exhibit hallmark AD pathologies, such as amyloid beta (Aβ) plaques and phosphorylated tau (pTau) tangles.^4^, ^5^

According to established neuropathological frameworks, the OC and OB are affected at relatively early disease stages; tau pathology is detectable from Braak stages I–II, while amyloid involvement corresponds to intermediate Thal phases (2–3) and moderate Consortium to Establish a Registry for Alzheimer's Disease scores.^6^, ^7^, ^8^, ^9^ Despite this early vulnerability, the olfactory system remains underrepresented in mechanistic AD research, which has largely focused on the hippocampus and neocortex.^10^

The OB is anatomically and functionally distinct from central limbic structures. It supports ongoing postnatal neurogenesis, maintains high metabolic demand, and receives direct synaptic input from olfactory sensory neurons that are exposed to the external environment via the nasal epithelium.^11^, ^12^, ^13^ While these features support sensory plasticity, they may also render the OB more susceptible to early degeneration.^14^, ^15^ Our previous work revealed severe histoarchitectural disruption in the OB glomerular layer (GL) in AD, suggesting early structural compromise.^16^ However, the cellular drivers of this vulnerability, including glial engagement and co‐pathologies with disease proteinopathies, remain poorly defined in humans.

Growing evidence indicates that neuroinflammation is not simply a consequence of AD pathology but actively contributes to disease progression.^17^, ^18^ Microglia and astrocytes undergo dynamic phenotypic changes in response to Aβ and pTau, exhibiting significant spatial and temporal heterogeneity.^19^, ^20^, ^21^, ^22^, ^23^ The OB has recently gained recognition as a neuroimmune interface, where glial cells not only surveil for pathology but also regulate local neurogenesis, synaptic remodeling, and neuronal survival.^24^, ^25^, ^26^

Apolipoprotein E (*APOE*) adds a further layer of complexity. As the strongest genetic risk factor for sporadic AD, the *APOE* ε4 allele influences glial phenotypes, impairs Aβ clearance, and promotes tau pathology.^27^, ^28^, ^29^ Beyond its genetic role, misfolded ApoE protein has been detected within plaques and glial inclusions in AD brains,^30^, ^31^ yet its spatial distribution and pathological impact within olfactory structures remain undercharacterized.

Herein, we present a comprehensive histopathological analysis of the human OC and OB across the clinical continuum of AD. Using immunohistochemistry and immunofluorescence on *post mortem* tissue, we examined Aβ, pTau, and apoE protein deposition alongside associated glial responses in cases ranging from cognitively normal (CN) to mild cognitive impairment (MCI) and AD. To further understand how these markers interact at a systems level, we conducted multivariate co‐expression analyses to assess coordinated changes in glial and pathological burden across regions and disease stages. Our objective was to determine how regional glial activation and protein burden evolved in the OC and OB and whether these processes unfold with distinct temporal and spatial dynamics. By integrating regional comparisons across clinical stages, this study provides novel insights into the selective vulnerability of olfactory areas and the spatial organization of glial pathology relationships.

**RESEARCH IN CONTEXT**

1. **Systematic review**: We reviewed prior literature using PubMed and Google Scholar to assess AD pathology in the olfactory system, with emphasis on glial responses and region‐specific vulnerability. While early Aβ and pTau accumulation in the OB and OC is well established, few studies have examined cell‐type‐specific glial pathology interactions across disease stages in human olfactory regions.
2. **Interpretation**: We show progressive protein accumulation and regionally distinct glial activation in the OC and OB. ApoE signal increases with disease stage and overlaps with Aβ and pTau, while co‐expression analyses reveal differing glial pathology network structures across regions.
3. **Future directions**: Future work should investigate how regional glial states contribute to olfactory vulnerability and functional decline, ideally using spatial and longitudinal approaches to clarify glial roles in early AD progression.

## 2 METHODS AND MATERIALS

### 2.1 Human *post mortem* brain tissue

Formalin‐fixed paraffin‐embedded (FFPE) brain tissue blocks containing the OC and OB were obtained from the Netherlands Brain Bank (NBB).^32^, ^33^ The NBB is a long‐established resource that applies standardized procedures for donor recruitment, clinical characterization, autopsy, and neuropathological documentation, ensuring consistent diagnostic classification across cohorts.^34^, ^35^, ^36^ All tissue blocks were collected and processed under uniform NBB protocols.^37^

The sample set included patients diagnosed with MCI (OC: *n* = 6, OB: *n* = 6), AD (OC: *n* = 11, OB: *n* = 12), and CN controls (OC: *n* = 5, OB: *n* = 6), selected to minimize demographic imbalances in age and sex at the group level (Table 1). Within the AD group, Braak neurofibrillary tangle staging was evenly represented, comprising four cases each at stages IV, V, and VI, consistent with National Institute on Aging‐Alzheimer's Association (NIA‐AA) guidelines for neuropathologic assessment.^38^ Informed consent for autopsy and the use of brain tissue for research purposes was obtained from donors or their next of kin by the NBB.

**TABLE 1:** Demographic and neuropathological characteristics of all human *post mortem* subjects.

Study ID	Group	Braak stage	*APOE* allele status^a^	Sex^b^	Age (years)	Tissue information	PMD (hours)	Thal score (0–3)	CERAD score (0–3)	Note
Case C01	CN	0	4/4	M	71	OB, OC	5:05	–	–	ARTAG
Case C04	CN	2	2/2	M	77	OB, OC	8:45	1	0	
Case C03	CN	2	3/3	F	71	OB	6:15	1	0	
Case C05	CN	2	3/3	F	87	OB, OC	4:35	2	0	
Case C02	CN	2	4/3	M	72	OB, OC	4:20	1	0	
Case C06	CN	2	4/3	F	81	OB, OC	7:40	1	0	
Case M02	MCI	1	3/3	F	76	OC	5:30	1	0	
Case M01	MCI	2	3/2	M	79	OB	6:30	1	0	
Case M03	MCI	2	3/3	M	84	OB, OC	3:30	1	0	ARTAG; non‐demented control with Lewy bodies
Case M04	MCI	2	3/3	F	88	OB, OC	7:25	–	–	PART
Case M05	MCI	3	3/3	M	85	OB, OC	5:10	2	1	PART; CAA
Case M07	MCI	3	3/3	F	88	OB, OC	4:30	1	0	ARTAG; PART
Case M06	MCI	3	4/2	F	85	OB, OC	7:05	3	2	ARTAG; CAA
Case A02	AD	4	3/3	F	94	OB	4:35	3	2	
Case A03	AD	4	4/3	F	83	OB, OC	4:30	3	2	CAA
Case A01	AD	4	4/3	M	86	OB, OC	3:45	3	2	ARTAG; CAA
Case A04	AD	4	4/3	M	95	OB, OC	8:25	3	2	ARTAG; CAA
Case A05	AD	5	3/3	M	85	OB, OC	6:10	3	3	ARTAG; CAA
Case A08	AD	5	3/3	F	88	OB, OC	5:55	3	3	ARTAG
Case A07	AD	5	3/3	M	95	OB, OC	6:05	3	2	
Case A06	AD	5	4/3	F	89	OB, OC	7:35	3	2	ARTAG; CAA
Case A12	AD	6	4/3	F	73	OB, OC	5:15	3	3	CAA
Case A09	AD	6	4/3	M	76	OB, OC	4:40	3	3	ARTAG; CAA
Case A11	AD	6	4/4	M	72	OB, OC	4:30	3	3	ARTAG
Case A10	AD	6	4/4	F	85	OB, OC	7:20	3	3	CAA

*Note*: Individual‐level demographic, genetic, and neuropathological data for all included cases, categorized by clinical diagnosis. All brain samples were FFPE tissue blocks from the OC and/or OB, obtained from the Netherlands Brain Bank (NBB). Clinical diagnoses were based on NBB standardized retrospective review of medical and neuropsychological records. Neuropathological assessments were performed by NBB‐certified neuropathologists. Braak neurofibrillary tangle stage, Thal Aβ phase score (0–3), and CERAD neuritic plaque score (0–3) are reported according to NIA–AA guidelines. “Note” summarizes relevant concomitant age‐associated or incidental pathologies (e.g., ARTAG, PART, CAA).

Abbreviations: AD, Alzheimer's disease; *APOE*, apolipoprotein E; ARTAG, aging‐related tau astrogliopathy; CAA, cerebral amyloid angiopathy; CERAD, Consortium to Establish a Registry for Alzheimer's Disease; CN, cognitively normal; FFPE, formalin‐fixed paraffin‐embedded; MCI, mild cognitive impairment; OB, olfactory bulb; OC, olfactory cortex; PART, primary age‐related tauopathy; PMD, *post mortem* delay.

^a^ *APOE* genotype based on ε2, ε3, ε4 alleles.

^b^ Sex coded as M = male, F = female.

All clinical diagnoses were confirmed according to the standardized assessment procedures of the NBB, based on retrospective review of medical and neuropsychological records by trained clinicians.^39^, ^40^, ^41^ MCI referred to cases showing objective cognitive decline greater than expected for age and education, without fulfilling diagnostic criteria for dementia, consistent with internationally accepted frameworks.^42^, ^43^ Detailed demographic information for all subjects is provided in Table 1.

The number of cases was determined by the availability of well‐characterized tissue blocks containing the OC and/or OB that met strict inclusion criteria, including high tissue integrity, short *post mortem* delay, a documented *ante mortem* clinical diagnosis, and a *post mortem* neuropathologic diagnosis assigned using established criteria.^38^, ^44^ Only donors with comprehensive medical documentation and without major comorbid neuropathology (e.g., Parkinson's disease, dementia with Lewy bodies, frontotemporal lobar degeneration, or progressive supranuclear palsy) were included. One MCI case was noted by the NBB as “non‐demented control with Lewy bodies”; this subject exhibited only incidental Lewy pathology without clinical Parkinsonism or dementia and was therefore retained in the study. Mild age‐associated changes such as aging‐related tau astrogliopathy (ARTAG), primary age‐related tauopathy (PART), or low‐grade cerebral amyloid angiopathy (CAA) were accepted, as they are common in elderly individuals and not expected to confound AD‐related olfactory pathology.

Whenever both regions were available, they were analyzed as complementary sites; however, in some donors only one region was suitable for histological assessment. Cases were selected to represent the AD continuum (CN, MCI, AD) with approximate age and sex matching across groups. Because of the limited availability of such high‐quality olfactory tissue, all eligible cases meeting these criteria were included.

### 2.2 Immunohistochemistry and image acquisition

#### 2.2.1 Double immunofluorescence staining

FFPE blocks were cut into 4‐µm‐thick sections, mounted on Superfrost Plus slides (VWR International), and dried overnight at 37°C. Sections were deparaffinized and rehydrated through graded xylene and ethanol, followed by rinsing in phosphate‐buffered saline (PBS). Antigen retrieval was performed by boiling sections in 10 mM citrate buffer (pH 6.0) for 15 min and cooling at room temperature (RT) for 30 min.

Endogenous peroxidase activity was blocked using 0.3% hydrogen peroxide in tris‐buffered saline (TBS) for 20 min. Non‐specific binding was blocked with 3% normal donkey serum (NDS) or bovine serum albumin (BSA) in TBS for 1 h at RT. Primary antibodies (Table 2) were applied overnight at 4°C. Following TBS‐Triton (TBS‐T) washes, sections were incubated with species‐specific Alexa Fluor‐conjugated secondary antibodies for 1 h at RT.

**TABLE 2:** List of antibodies.

Antibody	Manufacturer	Catalog number	Host	Dilution
Anti‐Aβ (6E10)	BioLegend	803001	Mouse	1:200
Anti‐Aβ (D54D2)	Cell signaling technology	8243	Rabbit	1:200
Anti‐pTau (Ser202, Thr205)	Invitrogen	MN1020	Mouse	1:200
Anti‐Iba1	Wako	019‐19741	Rabbit	1:200
Anti‐GFAP	Dako	Z0334	Rabbit	1:200
Anti‐apoE	Abcam	AB52607	Rabbit	1:100
Anti‐apoE	Abcam	AB1907	Mouse	1:100

*Note*: Antibody name, manufacturer, catalog number, host species, and dilution information are listed for all primary antibodies used in this study.

Abbreviations: Aβ, amyloid beta; apoE, apolipoprotein E; GFAP, glial fibrillary acidic protein; Iba1, ionized calcium‐binding adapter molecule 1; pTau, phosphorylated tau.

After PBS washes, nuclei were counterstained with Hoechst 33258 or DAPI (1 µg/mL, 5 min, RT), and lipofuscin autofluorescence was quenched using 0.1% Sudan Black B or TrueBlack™ Lipofuscin Autofluorescence Quencher (Biotium Inc.) in 70% ethanol for 10 min. Slides were washed and coverslipped with aqueous mounting medium (Immu‐Mount, Thermo Fisher Scientific). Slides were stored at 4°C for at least 48 h before imaging. Fluorescent images were acquired at Maastricht University using an Olympus BX51 microscope equipped with an Olympus DP70 digital camera and at Daegu Gyeongbuk Institute of Science and Technology (DGIST) using a Zeiss LSM780 confocal microscope. Images were processed with ImageJ/Fiji (version 1.54p; National Institutes of Health, Bethesda, MD, USA).

To verify the specificity of apoE immunostaining, we conducted additional validation using a second independently generated apoE antibody (Abcam, AB1907), in addition to the primary antibody used throughout the main analysis (Abcam, AB52607). Both antibodies have been applied in human AD neuropathology studies and exhibit the characteristic aggregate‐associated apoE pattern described in the literature.^45^ The second antibody produced the same plaque‐associated distribution and stage‐dependent increase observed with the primary antibody. Representative images and quantitative comparisons are provided in Figure S1.

#### 2.2.2 DAB immunohistochemistry

For DAB‐based immunohistochemistry, sections were prepared as above. After deparaffinization and antigen retrieval (10 mM citrate buffer, pH 6.0), endogenous peroxidase activity was quenched with 0.3% hydrogen peroxide in TBS. Non‐specific binding was blocked with 3% NDS or BSA.

Primary antibodies were applied overnight at 4°C. After washing in TBS‐T, sections were incubated with biotinylated secondary antibodies (1:200, 1 h, RT), followed by the Avidin‐Biotin Complex (ABC reagent, Vector Laboratories, 2 h, RT). DAB substrate solution (Sigma‐Aldrich) was applied for 3 to 10 min until signal developed. After dehydration in graded alcohol and xylene, slides were coverslipped using Entellan mounting medium (Merck). Brightfield images were acquired using the Olympus BX51 microscope.

### 2.3 Data analysis

#### 2.3.1 Image analysis

All quantitative analyses were based on immunofluorescence images. DAB‐stained sections were used for visualization only and were not included in quantitative measurements. For Aβ, pTau, Iba1, and glial fibrillary acidic protein (GFAP), four anatomically matched tissue sections per case were analyzed, and four non‐overlapping regions of interest (ROIs) were acquired from each section, yielding 16 ROIs per marker per case. For apoE, three matched sections were analyzed with four ROIs each, yielding 12 ROIs per case. ROIs were selected systematically from predefined anatomical landmarks within the OC and OB to ensure consistent sampling across individuals. In the OC, ROIs were selected from Layer I (one ROI), Layer II (two ROIs), and Layer III (one ROI). In the OB, ROIs were selected from the anterior olfactory nucleus (AON) (one ROI), granule cell layer (GCL) (one ROI), external plexiform layer (EPL) (one ROI), and GL (one ROI). Representative schematics of ROI placement are shown in Figure S2. All image analyses were conducted with diagnostic status blinded to the observer.

Quantification was performed in ImageJ/Fiji (version 1.54p). The fluorescence channel corresponding to each marker was extracted and converted to 8‐bit grayscale. A marker‐specific global threshold was determined from a representative training subset of images to establish reliable separation of signal from background. This fixed threshold was applied uniformly to all images for that marker. The percentage of positively stained area within each ROI was calculated using the Analyze Particles function. This percentage‐positive area represents the fraction of thresholded signal pixels relative to total ROI area; consequently, all group comparisons reflect absolute percentage‐area differences rather than values normalized to an external reference. Final values represent the mean percentage of positive areas across all ROIs for each marker per case.

#### 2.3.2 Microglial morphology analysis

Microglial morphology was assessed manually on single‐plane immunofluorescence images. Cells were classified into three categories using established criteria: (1) ramified, defined by small somata with multiple long, thin processes; (2) hypertrophic, defined by enlarged somata with thickened or retracted processes; and (3) amoeboid, defined by rounded somata with minimal or absent processes.^46^, ^47^ Only Iba1‐positive cells with clearly defined somata and primary processes within the focal plane were included. A minimum of 30 microglia per case was analyzed across the ROIs. Morphological scoring was performed independently by two blinded analyzers, and discrepancies were resolved by consensus. Automated skeletonization or Sholl‐based digital morphometry was not applied.

#### 2.3.3 Astrocyte morphology analysis

Astrocyte morphology was assessed on single‐plane immunofluorescence images. Soma size was quantified by manual tracing of GFAP‐positive cell bodies in ImageJ using the freehand selection tool, and the cross‐sectional area (µm^2^) was calculated from each traced outline. Only somata that were fully contained within the field and clearly delineated from adjacent structures were included. A minimum of 30 astrocytes per case were measured across the ROIs. For quantitative analyses, the mean soma area per case was used for comparisons between CN, MCI, and AD groups. Measurements were performed independently by two blinded analyzers, and discrepancies were resolved by consensus. No algorithmic segmentation was applied.

#### 2.3.4 Regional comparison score (OC/OB score) calculation

To compare regional differences in pathological and glial burden, we calculated a normalized OC/OB comparison score for each marker. This metric quantified the relative distribution of expression between the OC and OB within the same individual. For each marker and region, percentage‐positive area was computed per ROI and summarized to a single per‐case regional value as the mean across ROIs (“Methods,” Section 2.3.1); these per‐case regional means were used as the OC and OB inputs to the OC/OB formula. The score was defined as follows:

$$\mathrm{OC}/\mathrm{OB}\mathrm{score}\;=\frac{\left(\mathrm{OC}\;\mathrm{value}-\mathrm{OB}\;\mathrm{value}\right)}{\left(\mathrm{OC}\;\mathrm{value}+\mathrm{OB}\;\mathrm{value}\right)}$$

This normalization constrained values between −1 and +1. Positive values indicated proportionally higher burden in the OC, and negative values indicated proportionally higher burden in the OB. A score near zero indicated a balanced distribution across the two regions. We applied this calculation to Aβ, pTau, Iba1, GFAP, and apoE expression values. The score served as a within‐subject index of regional bias and enabled qualitative assessment of shifts in regional distribution across clinical groups.

#### 2.3.5 Statistical analysis

Statistical analyses and data visualization were conducted using GraphPad Prism version 9.0 (GraphPad Software, USA). For comparisons across clinical groups, one‐way analysis of variance (ANOVA) followed by Tukey's post hoc test was applied. Unpaired, two‐tailed Student's *t*‐tests were used for genotype or region‐based comparisons. A *p* value < 0.05 was considered statistically significant. Statistical significance in figures is indicated as follows: *p < 0.05, **p < 0.01, ***p < 0.001, ****p < 0.0001. Associations of age and sex with primary outcomes were assessed using Spearman's rank correlation (age) and the Mann–Whitney U test (sex) (Table S1). Sensitivity analyses repeated the primary one‐way ANOVA after excluding the oldest AD case and the youngest CN case within each region (Table S2).

#### 2.3.6 Co‐expression analysis

To evaluate whether different disease stages involve coordinated changes across multiple biological markers rather than isolated shifts in single variables, we conducted correlation analyses among Aβ, pTau, Iba1, GFAP, and apoE expression levels. Pearson's correlation coefficients were calculated for normally distributed data, and Spearman's rank correlation coefficients were used for non‐normally distributed variables. Marker expression values were organized into pivot tables per subject, and region‐specific correlation matrices (OC and OB) were generated. All available cases across diagnostic groups (CN, MCI, and AD) were included in each regional correlation matrix. These matrices were visualized as heatmaps using Python (Pandas, NumPy, Seaborn, and Matplotlib libraries) to summarize shared expression patterns, intermarker associations, and region‐specific differences in co‐expression networks^48^, ^49^, ^50^, ^51^ (documentation accessed June 27, 2025).

## 3 RESULTS

### 3.1 Aβ and pTau accumulation in OC and OB

**FIGURE 1:**
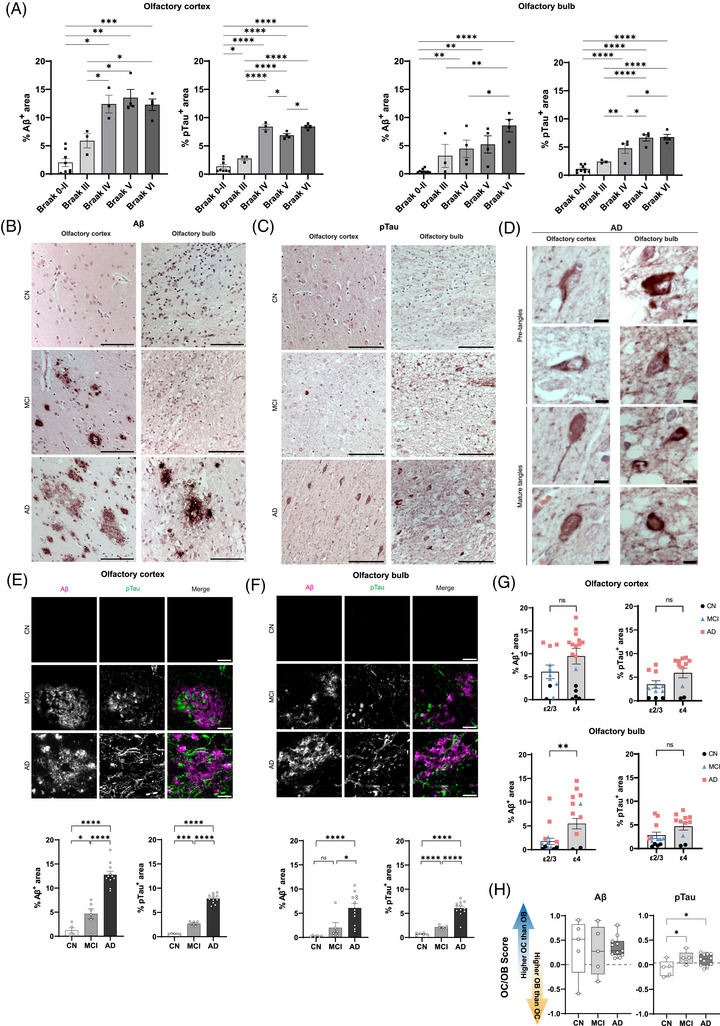
Progressive accumulation of Aβ and pTau pathology in the OC and OB. (A) Quantitative analysis of Aβ‐ and pTau‐positive area in OC and OB across Braak stages. (B and C) Representative DAB‐stained sections showing Aβ plaques and pTau‐positive structures in both regions. (D) High‐magnification image highlighting pTau pretangles and mature neurofibrillary tangles in OC and OB. (E and F) Double immunofluorescence quantification of Aβ and pTau burden across clinical groups (CN, MCI, AD). (G) Comparison of Aβ and pTau burden by *APOE* genotype (ε2/ε3 vs ε4) in OC and OB. (H) Normalized OC/OB burden scores for Aβ and pTau across clinical groups. Statistics: one‐way ANOVA with Tukey's post hoc test for multigroup comparisons; unpaired two‐tailed *t*‐test for two‐group comparisons; *p* < 0.05 considered significant. Scale bars: (B), (C) 1000 µm; (D) 10 µm; (E), (F) 20 µm. Aβ, amyloid beta; AD, Alzheimer's disease; *APOE*, apolipoprotein E; CN, cognitively normal; MCI, mild cognitive impairment; OB, olfactory bulb; OC, olfactory cortex; pTau, phosphorylated tau.

To validate pathological staging and assess tissue quality, we examined the relationship between Braak stage and proteinopathy burden in the OC and OB. Quantitative analysis showed that Aβ and pTau deposition increased from Braak 0 to II and Braak III to IV in both regions (Aβ: OC *p* < 0.01, OB *p* < 0.001; pTau: OC *p* < 0.0001, OB *p* < 0.0001; Figure 1A). Beyond Braak IV, values approached a plateau, and no significant differences were observed between Braak V and VI. In the OC, pTau showed a modest decrease from Braak IV to Braak V, although the distributions overlapped substantially and remained within a similar overall range across Braak IV to VI. These results demonstrate steep increases through early and intermediate Braak stages, with limited change between Braak V and VI.

Qualitative evaluation of DAB‐stained sections confirmed the presence of Aβ plaques and pTau tangles in both regions. Notably, pretangles and mature neurofibrillary tangles were observed, particularly in the pTau‐stained sections of both OB and OC (Figure 1B–D).

To quantify pathology burden, we conducted double immunofluorescence staining for Aβ and pTau. In the OC, the percentage of Aβ‐positive area was significantly increased in MCI compared to CN (*p* < 0.05) and further elevated in AD (*p* < 0.0001). Similarly, pTau burden rose significantly from CN to MCI (*p* < 0.001) and from MCI to AD (*p* < 0.0001) (Figure 1E).

In the OB, Aβ burden was not significantly elevated in MCI compared to CN but showed a robust increase in AD compared to both CN (*p* < 0.0001) and MCI (*p* < 0.05) (Figure 1F). The absence of detectable Aβ signal in MCI in the OB was also visible in DAB‐stained sections (Figure 1B). In contrast, pTau accumulation followed a strong stepwise pattern, with significant increases between CN and MCI (*p* < 0.0001) and from MCI to AD (*p* < 0.0001) (Figure 1F).

We next stratified Aβ and pTau burden by *APOE* ε4 carrier status to examine genotype‐associated patterns. In the OC, neither Aβ nor pTau burden differed significantly between *APOE* Aε2/ε3 and *APOE* ε4 carriers, although *APOE* ε4‐positive individuals exhibited greater variability, particularly among AD cases (Figure 1G). Because *APOE* ε4 carriers were concentrated within the AD group, distributions largely reflected underlying clinical‐stage composition. In the OB, Aβ burden appeared higher in *APOE* ε4 carriers compared to *APOE* ε2/ε3 individuals (*p* < 0.01; Figure 1G), although this difference corresponded to group composition. pTau levels in the OB showed a modest upward shift in *APOE* ε4 carriers but overlapped substantially with *APOE* ε2/ε3 distributions.

To assess regional distribution of pathological protein accumulation, we calculated normalized OC/OB comparison scores for Aβ and pTau (Figure 1H). For Aβ, scores were predominantly positive across all groups, indicating greater amyloid burden in the OC relative to the OB. No significant group differences were detected. For pTau, scores were higher in MCI and AD compared to CN (*p* < 0.05), without differences between MCI and AD.

Taken together, these results show increases in both Aβ and pTau burden across clinical stages in the OC and OB, with differing magnitudes across regions.

### 3.2 Microglial activation in OC and OB

**FIGURE 2:**
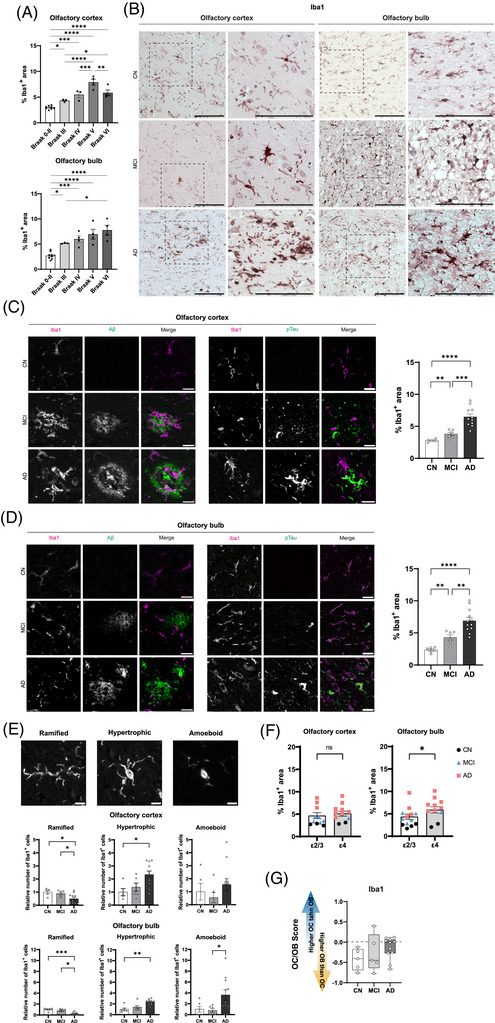
Progressive and region‐specific microglial activation in OC and OB. (A) Quantitative analysis of Iba1 immunoreactivity across Braak stages in OC and OB. (B) Representative DAB‐stained sections from CN, MCI, and AD cases in OC and OB; insets show higher‐magnification views of microglial morphology. (C and D) Quantification of Iba1 burden across clinical groups (CN, MCI, AD) in OC (C) and OB (D), with double immunofluorescence showing Iba1‐positive microglia surrounding Aβ plaques and pTau‐positive structures. (E) Classification of microglial morphology into ramified, hypertrophic, and amoeboid subtypes with representative images and quantification of relative number of each subtype across clinical groups. (F) Comparison of Iba1 burden by *APOE* genotype (ε2/ε3 vs ε4) in OC and OB. (G) Normalized OC/OB Iba1 burden scores across clinical groups. Statistics: one‐way ANOVA with Tukey's post hoc test for multigroup comparisons; unpaired two‐tailed *t*‐test for two‐group comparisons; *p* < 0.05 considered significant. Scale bars: (B) 100 µm; inset images in (B) 20 µm; (C), (D) 20 µm; (E) 10 µm. AD, Alzheimer's disease; *APOE*, apolipoprotein E; CN, cognitively normal; Iba1, ionized calcium‐binding adapter molecule 1; MCI, mild cognitive impairment; OB, olfactory bulb; OC, olfactory cortex; Aβ, amyloid beta; pTau, phosphorylated tau.

To characterize neuroinflammatory changes across the AD continuum, we evaluated microglial activation in the OC and OB using Iba1. Quantitative analysis revealed higher Iba1‐positive area across Braak stages, with significantly elevated microglial burden in both regions from Braak stage III onward (*p* < 0.0001; Figure 2A). Qualitative DAB‐stained sections showed higher microglial density, larger somata, and thicker processes in AD compared to CN and MCI (Figure 2B).

To explore the spatial relationship between microglia and pathological proteins, we performed double immunofluorescence staining for Iba1 with Aβ and pTau. In both regions, Iba1‐positive microglia closely apposed to and interdigitated with Aβ‐positive plaques, consistent with plaque‐associated microglial engagement observed in human AD tissue (Figure 2C,D). Quantitative analysis across clinical groups confirmed a stage‐dependent increase in Iba1 burden in both regions. In the OC, Iba1 expression was significantly higher in MCI compared to CN (*p* < 0.01) and further elevated in AD (*p* < 0.001 vs MCI; *p* < 0.0001 vs CN; Figure 2C). In the OB, a similar trend was observed, with significant increases from CN to MCI (*p* < 0.01) and from MCI to AD (*p* < 0.01; *p* < 0.0001 vs CN; Figure 2D).

To further resolve morphology, we classified microglia into ramified, hypertrophic, and amoeboid subtypes (Figure 2E). In the OC, ramified microglia were significantly reduced in AD (*p* < 0.05), and hypertrophic microglia were increased in AD compared to CN (*p* < 0.05). In the OB, ramified microglia were significantly reduced in AD compared to CN (*p* < 0.001) and MCI (*p* < 0.05). Hypertrophic microglia were elevated in AD (*p* < 0.01 vs CN), and amoeboid microglia were significantly increased in AD compared to CN (*p* < 0.05). Iba1 values overlapped between *APOE* ε2/ε3 and *APOE* ε4 carriers in both regions (Figure 2F). *APOE* ε4 carriers showed wider variability due to group composition. Normalized OC/OB comparison scores for Iba1 (Figure 2G) were predominantly negative in CN, closer to zero in MCI, and near zero in AD. No significant group differences were detected.

Taken together, these data show differences in Iba1‐positive area and microglial morphology across clinical stages in both regions.

### 3.3 Astrocytic activation in OC and OB

**FIGURE 3:**
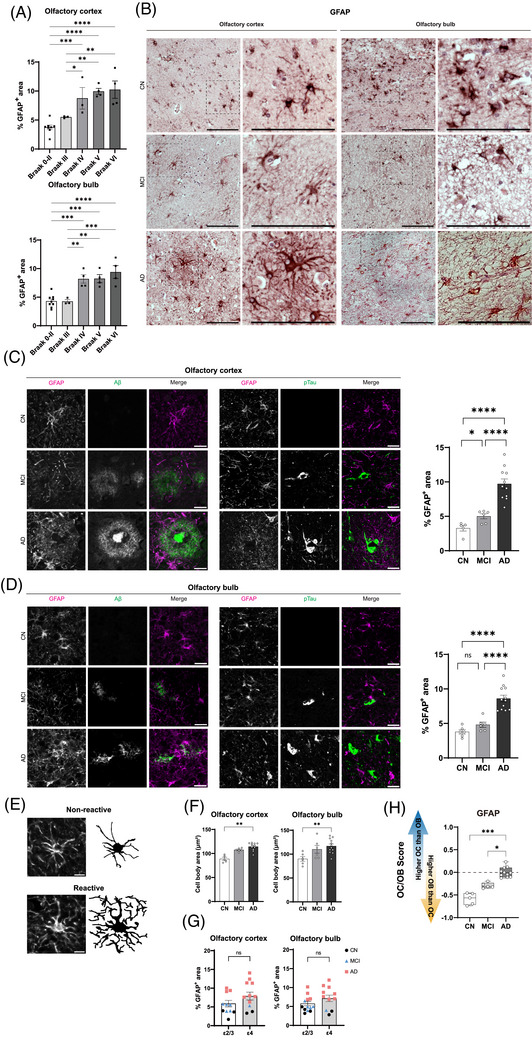
Astrocyte activation and regional distribution across AD stages in OC and OB. (A) Quantitative analysis of GFAP immunoreactivity across Braak stages in OC and OB. (B) Representative DAB‐stained sections from CN, MCI, and AD cases, with insets showing higher‐magnification views of astrocyte morphology. (C and D) Double immunofluorescence images showing GFAP with Aβ and pTau in OC (C) and OB (D) across clinical groups. (E) Schematic and representative images of non‐reactive and reactive astrocyte morphologies. (F) Quantification of astrocyte cell body area in OC and OB across clinical groups. (G) Comparison of GFAP burden by *APOE* genotype (ε2/ε3 vs ε4) in OC and OB. (H) Normalized OC/OB GFAP burden across clinical groups. Statistics: one‐way ANOVA with Tukey's post hoc test for multigroup comparisons; unpaired two‐tailed *t*‐test for two‐group comparisons; *p* < 0.05 considered significant. Scale bars: (B) 100 µm; insets in (B) 20 µm; (C), (D) 20 µm; (E) 10 µm. Aβ, amyloid beta; AD, Alzheimer's disease; *APOE*, apolipoprotein E; CN, cognitively normal; GFAP, glial fibrillary acidic protein; MCI, mild cognitive impairment; OB, olfactory bulb; OC, olfactory cortex; pTau, phosphorylated tau.

To examine astrocytic responses across the disease continuum, we assessed GFAP immunostaining in the OC and OB. Quantitative analysis revealed a significant stage‐dependent increase in GFAP‐positive area in both regions, with marked elevation from Braak stage III (*p* < 0.0001; Figure 3A).

DAB‐stained sections showed higher astrocyte density and hypertrophic morphology in AD compared to CN and MCI in both regions (Figure 3B).

We next investigated the spatial association between astrocytes and Aβ/pTau pathology using double immunofluorescence staining of GFAP with Aβ and pTau in OC and OB (Figure 3C,D). In CN, astrocytes were sparse and showed minimal overlap with Aβ or pTau. In MCI and AD, GFAP immunoreactivity increased markedly, with astrocytes closely surrounding Aβ plaques and pTau‐positive neurons, especially in AD. Quantification showed a significant increase in GFAP expression in the OC beginning in MCI (Figure 3C; *p* < 0.05 vs CN) and a further rise in AD (*p* < 0.0001 vs both CN and MCI). In the OB, significant astrocyte activation was observed only in AD (Figure 3D; *p* < 0.0001 vs CN and MCI), with no difference between CN and MCI.

To further assess reactivity, we evaluated astrocyte morphology by categorizing cells as non‐reactive (thin processes and small cell bodies) or reactive (hypertrophic cell bodies with thick, branched processes; Figure 3E). We quantified astrocyte cell body size as an index of hypertrophy. In the OC, cell body area was significantly larger in AD than CN (*p* < 0.01; Figure 3F). In the OB, cell body size was also significantly greater in AD compared to CN (*p* < 0.01), with no significant differences between CN and MCI or between MCI and AD (Figure 3F). To examine whether *APOE* genotype influenced astrocytic activation, we descriptively compared GFAP‐positive area between *APOE* ε2/ε3 and *APOE* ε4 carriers (Figure 3G). GFAP values overlapped extensively between genotypes in both regions, and *APOE* ε4 carriers showed greater variability primarily because a larger proportion of *APOE* ε4 carriers belonged to the AD group.

Finally, to examine regional redistribution of astrocyte activation, we calculated normalized OC/OB comparison scores for GFAP (Figure 3H). In CN individuals, scores were significantly negative, indicating greater GFAP expression in the OB. Scores increased toward zero in MCI and became significantly positive in AD, indicating a gradual shift in regional GFAP expression. Statistical analysis confirmed a significant increase from CN to MCI (*p* < 0.001) and from MCI to AD (*p* < 0.05).

Together, these findings highlight a stage‐dependent increase in astrocyte reactivity, with earlier involvement in the OC, later hypertrophic changes in both regions, and a shift in GFAP expression from higher OB than OC in CN to higher OC than OB in AD.

### 3.4 apoE protein expression in OC and OB

**FIGURE 4:**
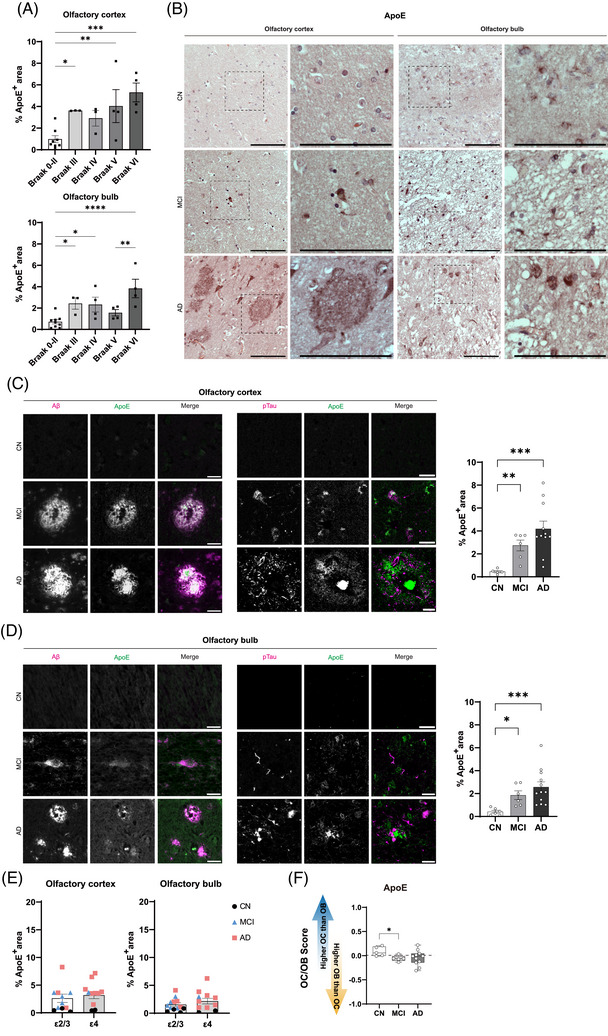
apoE immunoreactivity and spatial colocalization with Aβ and pTau in OC and OB. (A) Quantitative analysis of apoE immunoreactivity across Braak stages in OC and OB. (B) Representative DAB‐stained sections from CN, MCI, and AD cases. apoE‐positive cells in MCI and AD appear more numerous and more intensely labeled, with granular perinuclear patterns; insets show higher‐magnification views. (C and D) Double immunofluorescence images of apoE with Aβ and pTau in OC (C) and OB (D) in across clinical groups. (E) Comparison of apoE‐positive area between *APOE* ε2/ε3 and *APOE* ε4 carriers in OC and OB. (F) Normalized OC/OB apoE burden scores across clinical groups. Statistics: one‐way ANOVA with Tukey's post hoc test for multigroup comparisons; unpaired two‐tailed *t*‐test for two‐group comparisons; *p* < 0.05 considered significant. Scale bars: (B) 100 µm; insets in (B) 20 µm; (C), (D) 20 µm. Aβ, amyloid beta; AD, Alzheimer's disease; apoE, apolipoprotein E; CN, cognitively normal; MCI, mild cognitive impairment; OB, olfactory bulb; OC, olfactory cortex; pTau, phosphorylated tau.

To investigate regional apoE pathology, we assessed ApoE protein expression in the OC and OB. In both regions, apoE expression increased significantly across Braak stages in both regions (*p* < 0.01; Figure 4A), reflecting stage‐dependent increases in detectable apoE signal.

DAB staining supported these results, showing a stage‐related increase in apoE immunoreactivity across clinical stages (Figure 4B). In CN cases, apoE was sparse and faint. Qualitative analysis revealed that in MCI and AD cases, apoE‐positive cells appeared more abundant and intensely labeled, often displaying granular, perinuclear patterns (Figure 4B).

To assess spatial relationships with AD pathology, we performed double immunofluorescence staining of apoE with Aβ and pTau in OC and OB (Figure 4C,D). In CN cases, the minimal and sparse presence of apoE, Aβ, and pTau signal precluded definitive colocalization analysis. Despite the low signal intensity, quantification of the CN group was conducted to establish a baseline threshold against which the stage‐dependent protein accumulation in MCI and AD could be compared. In MCI and AD, however, apoE showed a close spatial association with Aβ plaques and an intermixed distribution adjacent to pTau‐positive structures, forming dense, aggregated deposits within or surrounding Aβ plaque cores and in proximity to pTau‐labeled neurons. Quantification confirmed a significant increase in apoE‐positive area in MCI versus CN (*p* < 0.01 in OC, *p* < 0.05 in OB) and a further increase in AD (*p* < 0.001 for both regions).

To examine whether *APOE* genotype influenced apoE protein levels, we compared apoE‐positive areas between *APOE* ε2/ε3 and *APOE* ε4 carriers (Figure 4E). In this cohort, apoE burden appeared comparable between genotype groups in both the OC and OB. *APOE* ε4 carriers showed wider variability, reflecting the larger proportion of *APOE* ε4 carriers within the AD group. To further support staining specificity, we replicated apoE immunohistochemistry using a second commercially validated apoE antibody, which reproduced the same aggregate‐associated pattern observed with the primary antibody (Figure S1).

Finally, to assess regional distribution, we calculated OC/OB comparison scores for apoE across clinical groups (Figure 4F). In CN, scores were significantly positive, indicating greater apoE burden in the OC. Across disease stages, scores shifted toward zero, with a significant reduction from CN to MCI (*p* < 0.05).

These results collectively outline a stage‐dependent increase in apoE burden, its spatial proximity to pathological proteins, and a shift toward regionally balanced expression with advancing disease.

### 3.5 Co‐expression of pathological and glial markers reveals region‐specific network dynamics

**FIGURE 5:**
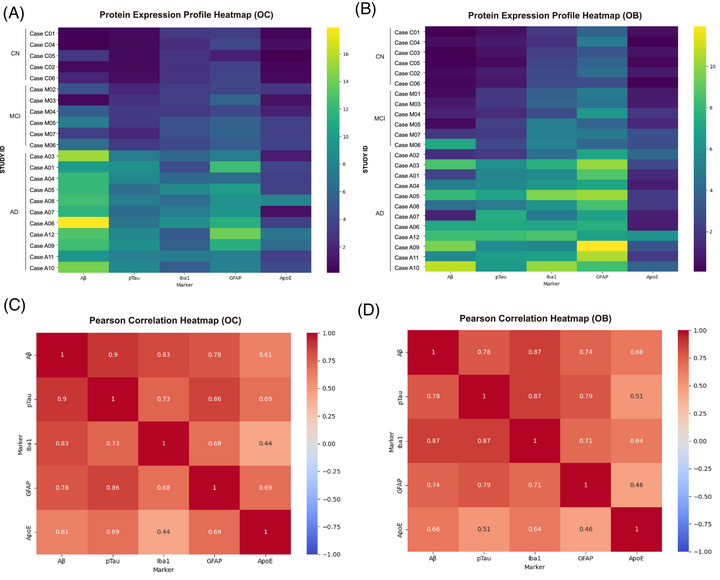
Region‐specific multivariate expression and intermarker correlations in OC and OB. (A and B) Protein expression profile heatmaps for Aβ, pTau, Iba1, GFAP, and ApoE across individual cases in OC (A) and OB (B), arranged by clinical groups (CN, MCI, AD). (C and D) Pearson correlation heatmaps for Aβ, pTau, Iba1, GFAP, and apoE in OC (C) and OB (D). Correlation analyses included all available cases (CN, MCI, and AD) within each region. Statistics: Pearson's correlation coefficients were used for normally distributed variables; Spearman's rank correlation was used for non‐normal distributions. Correlation matrices were generated per region (OC and OB), with *p* < 0.05 considered significant. Aβ, amyloid beta; AD, Alzheimer's disease; apoE, apolipoprotein E; CN, cognitively normal; GFAP, glial fibrillary acidic protein; Iba1, ionized calcium‐binding adapter molecule 1; MCI, mild cognitive impairment; OB, olfactory bulb; OC, olfactory cortex; pTau, phosphorylated tau.

To investigate region‐specific expression dynamics and intermarker interactions, we examined subject‐level immunoreactivity for five key markers – Aβ, pTau, Iba1, GFAP, and apoE – within the OC and OB, as visualized in the heatmaps (Figure 5A,B). In the OC (Figure 5A), AD cases consistently exhibited high expression levels of Aβ, pTau, Iba1, and GFAP, while MCI cases presented intermediate expression and CN individuals showed low levels across all markers.

In the OB (Figure 5B), AD cases displayed elevated GFAP and Iba1 expression, with a moderate increase in pTau. However, Aβ expression was more heterogeneous among AD individuals, indicating region‐specific variability in amyloid pathology. MCI cases again showed intermediate levels, while CN cases had uniformly low expression. Notably, apoE levels remained relatively low in both regions and demonstrated minimal contrast between groups in the OB.

To characterize the co‐expression architecture of these markers, Pearson correlation analyses were conducted (Figure 5C,D). In the OC (Figure 5C), Aβ and pTau were highly correlated (*r* = 0.90), indicating tight co‐regulation of amyloid and tau pathology. Strong associations were also observed between GFAP and pTau (*r* = 0.86) and Aβ and Iba1 (*r* = 0.83). ApoE exhibited moderate correlations with pTau (*r* = 0.69), GFAP (*r* = 0.69), and Aβ (*r* = 0.61).

In contrast, the OB (Figure 5D) displayed a distinct correlation structure. Iba1 emerged as a central hub, strongly correlating with both Aβ and pTau (*r* = 0.87), and GFAP showed robust associations with pTau (*r* = 0.79) and Aβ (*r* = 0.74). ApoE maintained relatively lower correlations in the OB, including *r* = 0.66 with Aβ and *r* = 0.64 with Iba1.

Collectively, these findings highlight both shared upregulation trends across disease stages and region‐specific co‐expression patterns. The OC exhibits a tightly integrated pathological‐glial signature, whereas the OB is characterized by more glia‐centric co‐expression, particularly involving Iba1.

## 4 DISCUSSION

The olfactory system is among the earliest regions affected in AD, yet its cellular and molecular pathology remains less thoroughly characterized than the hippocampus or neocortex.^1^, ^52^ We conducted a comprehensive histological analysis of the human OC and OB across the clinical continuum of AD. Our findings reveal a stage‐dependent, regionally distinct accumulation of pathological proteins and glial activation, underscoring spatiotemporal heterogeneity within the olfactory system. Across both regions, pathological burden increased steeply through Braak IV and plateaued between Braak V and VI, consistent with a plateau of Aβ and pTau accumulation dynamics at advanced stages. Tissue samples were rigorously validated for hallmark AD pathologies, providing a robust basis for quantitative comparisons.

We observed the expected increase in Aβ and pTau burden in both regions^5^, ^53^ but identified divergent glial responses. Microglia transformed from ramified to hypertrophic and amoeboid states, while astrocytes displayed reactive hypertrophic morphologies (Figures 2E and 3E,F). The OB's heightened vulnerability likely stems from unique anatomical properties, including lifelong neurogenesis, environmental exposure, high metabolic demand, and absence of a classical blood‐brain barrier,^10^, ^11^, ^13^, ^54^, ^55^, ^56^, ^57^ which collectively increase susceptibility to inflammation and metabolic perturbation. Glial changes were particularly pronounced in the OB, aligning with recent work showing OB amyloid burden increases from mid‐Thal phases and correlates with cognitive decline.^58^ Our prior work similarly revealed GL disorganization, indicating early structural deterioration.^16^, ^59^

Double immunofluorescence showed microglia and astrocytes closely associated with protein aggregates, suggesting engagement with local pathology rather than propagation.^60^, ^61^ Astrocytes adopt diverse reactive states,^62^ and astrocytic signaling can influence tau phosphorylation.^63^ Similarly, microglia in plaque‐associated regions frequently resemble disease‐associated phenotypes involved in compacting amyloid.^23^ This spatial convergence supports the view that glial activation reflects local microenvironmental changes in regions of high pathological load.^64^, ^65^

These findings reveal distinct temporal and regional glial patterns. Microglial activation (Iba1) was initially higher in CN individuals in the OB, consistent with early immune surveillance or homeostatic monitoring. Across disease stages, OC/OB Iba1 score moved toward zero, indicating reduced regional asymmetry. In contrast, astrocytic activation (GFAP) increased stage‐dependently in the OC, reversing the initial pattern of higher OB GFAP levels. Astrocytes exhibit regional molecular heterogeneity, shaping their responses to injury and pathological burden,^66^ aligning with transcriptomic evidence of region‐specific reactivity.^67^ Thus, microglial burden is initially higher in the OB but converges with OC levels in AD, whereas astrocytic reactivity increases progressively in the OC, eventually exceeding the OB.

Although amoeboid microglia proportions did not increase significantly, their higher frequency suggests a phenotypic transition toward reactive states. The overall pattern suggests a gradual rebalancing of glial activation rather than abrupt subtype‐specific shifts. Increased OB glial activation may reflect a microenvironment that becomes progressively less permissive to cellular maintenance and circuit stability, consistent with evidence that inflammation perturbs olfactory function.^68^, ^69^, ^70^, ^71^, ^72^, ^73^, ^74^, ^75^, ^76^

ApoE regulates lipid homeostasis and inflammatory tone, processes central to AD pathogenesis.^27^, ^28^, ^29^, ^30^, ^31^ We interpreted apoE signal as pathology‐associated, given its low detectability in controls. We observed stage‐dependent apoE accumulation in both regions, spatially associated with Aβ plaques and pTau structures. *APOE* genotype did not exert an independent effect on apoE burden; variability among *APOE* ε4 carriers reflected diagnostic distribution rather than genotype‐specific regulation.

Co‐expression network analysis further contextualized these findings. Heatmaps showed stage‐dependent increases in Aβ, pTau, Iba1, and GFAP, though relationships were regionally distinct. In the OC, markers were tightly correlated (pTau‐GFAP, *r* = 0.86; Aβ‐Iba1, *r* = 0.83), likely reflecting coordinated responses to shared regional environments rather than selective coupling to specific protein species. apoE exhibited moderate correlations with pTau (*r* = 0.69), GFAP (*r* = 0.69), and Aβ (*r* = 0.61), indicating secondary integration into the OC pathological network.

In contrast, the OB displayed a microglia‐centered co‐expression profile. Iba1 correlated strongly with both Aβ and pTau (*r* = 0.87), while GFAP correlated with pTau (*r* = 0.79) and Aβ (*r* = 0.74). ApoE correlations were lower (*r* = 0.66 with Aβ, *r* = 0.64 with Iba1), suggesting weaker integration into OB networks. These findings indicate that OC and OB pathology arises within distinct glial pathology network architectures, supported by spatial transcriptomics showing regional heterogeneity.^77^

Prior work showed *APOE* isoforms modulated microglial activation and plaque compaction in a context‐dependent manner.^78^, ^79^ However, we observed aberrant apoE protein aggregation in both the OC and OB regardless of genotype. Validation with an independent antibody supports the specificity of this staining, consistent with evidence that apoE aggregates act as cofactors in plaque formation and modulate both the toxicity and clearance of Aβ species.^80^, ^81^, ^82^, ^83^, ^84^

The strengths of this study include the parallel examination of the OC and OB and the integration of quantitative morphometry with genotype data. However, sample size and group composition imposed statistical constraints. As age and sex overlapped partially with diagnostic stage, covariate‐adjusted models (e.g., ANCOVA) were not statistically appropriate; nonetheless, observed group differences were not driven by extreme imbalances in these variables.

In the context of these constraints, our region‐specific patterns are consistent with distinct glial roles in tissue surveillance and circuit maintenance. The dynamic surveillance of microglia contrasts with the stationary, homeostatic functions of astrocytes.^85^, ^86^ These intrinsic differences likely drive the divergent activation profiles we observed, where microglial remodeling of synaptic architecture may further compromise OB network integrity and olfactory processing in AD.^87^ This pattern can be indicative of a more severe and actively progressive neurodegenerative process in the OB, characterized by sustained microglial recruitment relative to the OC. Such temporal advancement of pathology in the OB may place it chronologically ahead of cortical involvement, thereby maintaining a pathological state that persistently outpaces astrocytic mechanisms of homeostatic restoration.

Several methodological limitations warrant consideration. First, the cross‐sectional design precludes inference about temporal progression of glial or pathological changes. Second, *APOE* genotype was partially collinear with diagnostic stage (*APOE* ε4 carriers were more common in AD), preventing the isolation of independent genotype effects. Third, we used single canonical markers (Iba1, GFAP), which do not resolve transcriptionally defined glial subtypes or functional states. Fourth, given the inherent limitations of histology in FFPE tissue, our morphological classification relied on two‐dimensional images, limiting inference about three‐dimensional structural complexity. Fifth, global OB volume was not quantified due to histological trimming variability^88^; however, ROI‐based percentage‐positive area remains a robust, size‐independent proxy for local pathology. Finally, race/ethnicity and the interval from the last clinical examination to death were unavailable or limited in the source dataset and could not be evaluated as covariates.

In conclusion, the human olfactory system undergoes stage‐dependent, regionally distinct glial activation and protein accumulation across the AD continuum, with the OB displaying unique pathological features. By integrating morphometric, immunohistochemical, and correlation‐based analyses, we show that glial pathology relationships differ across olfactory regions: Astrocytic responses are more tightly aligned with tau pathology in the OC, while microglial activation is more strongly coupled with Aβ and pTau in the OB. These findings refine current models of AD propagation by incorporating anatomically distinct glial pathology networks within the olfactory system and highlight how glial phenotype and local protein accumulation shape spatially specific pathological trajectories. This systems‐level perspective underscores the olfactory system as a key site of early AD vulnerability and suggests that the divergent glial pathology architectures of the OC and OB may contribute uniquely to the onset and advancement of olfactory dysfunction and broader cognitive decline in AD. These insights may guide the development of targeted strategies for identifying and modulating region‐specific mechanisms across disease stages.

## CONFLICT OF INTEREST STATEMENT

The authors declare that they have no conflict of interest. Author disclosures are available in the supporting information.

## FUNDING INFORMATION

This research was supported by Basic Science Research Program through the National Research Foundation of Korea (NRF) funded by the Ministry of Education (RS‐2020‐NR049577) and the Ministry of Science and ICT (RS‐2023‐00278057).

## CONSENT STATEMENT

*Post mortem* human brain tissues were obtained from the NBB, in accordance with institutional and national ethical guidelines. Informed consent for autopsy and the use of brain tissue for research purposes was obtained by the NBB from all donors or their next of kin. All procedures were performed following ethical standards and approved protocols for human tissue research.

## Supporting information



Supporting Information

Supporting Information
