# Supplementary material for: Glial pathology networks reveal early olfactory vulnerability in post mortem human Alzheimer's disease
Source: Alzheimers Dement. 2026 Apr 6;22(4):e71322. doi: 10.1002/alz.71322 (PMC13053933; doi:10.1002/alz.71322)
Supplement: Supplementary file 1 — Supporting Information [file ALZ-22-e71322-s001.pdf]

Alzheimer's & Dementia®
THE JOURNAL OF THE ALZHEIMER'S ASSOCIATION

Supplementary Materials for

Glial–Pathology Networks Reveal Early Olfactory Vulnerability in Postmortem Human Alzheimer's Disease

Da Hae Jung, Eunji Park, Hyeon Chang Ju, Cheil Moon*, Ali Jahanshahi*

This PDF file includes:

Supplementary Figure 1 and 2

Supplementary Table 1 and 2

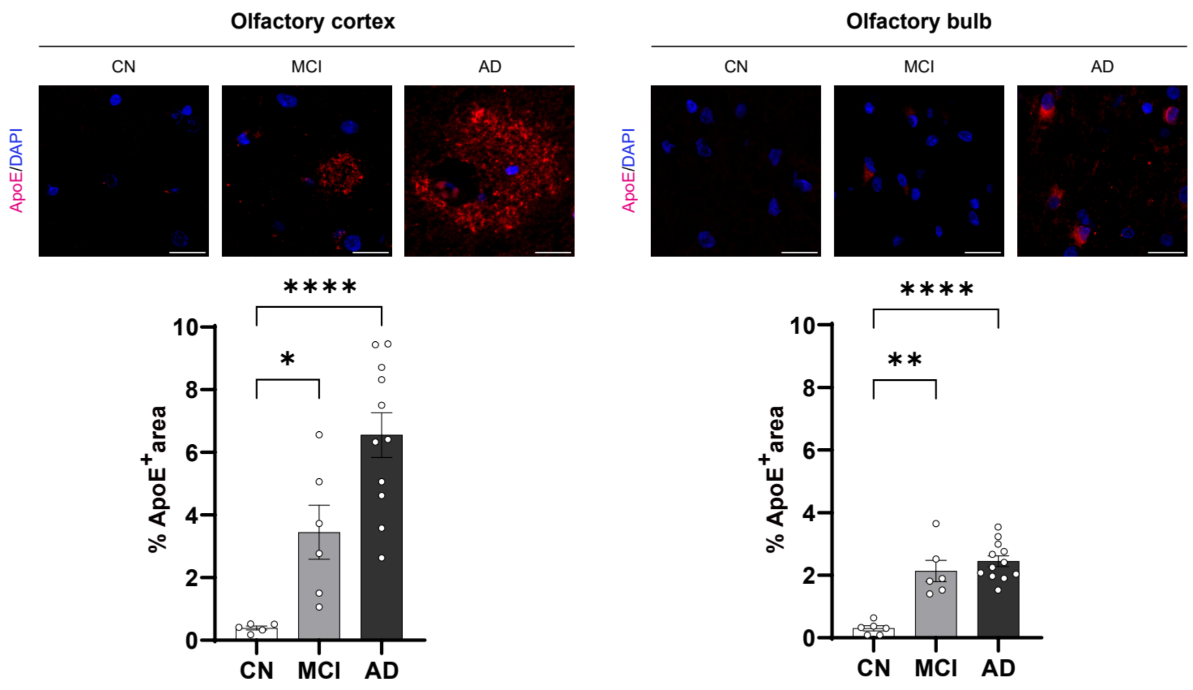

Supplementary Figure 1. ApoE immunoreactivity in the OC and OB using a second antibody.

Representative immunofluorescence images of ApoE (red) and DAPI (blue) in the OC (left) and OB (right) across CN, MCI, and AD cases. ApoE signal is minimal in CN and more prominent in MCI and AD, with granular aggregates visible at higher magnification. Quantification of ApoE-positive area was performed in ImageJ/Fiji (v1.54p) using the same thresholding and ROI-based pipeline described in Methods and is shown for OC and OB across clinical groups (OC: CN n = 5, MCI n = 6, AD n = 11; OB: CN n = 6, MCI n = 6, AD n = 12).

Statistics: One-way ANOVA with Tukey's post hoc test for multi-group comparisons; unpaired two-tailed t-test for two-group comparisons. $p < 0.05$ considered significant.

Scale bars: 10 µm.

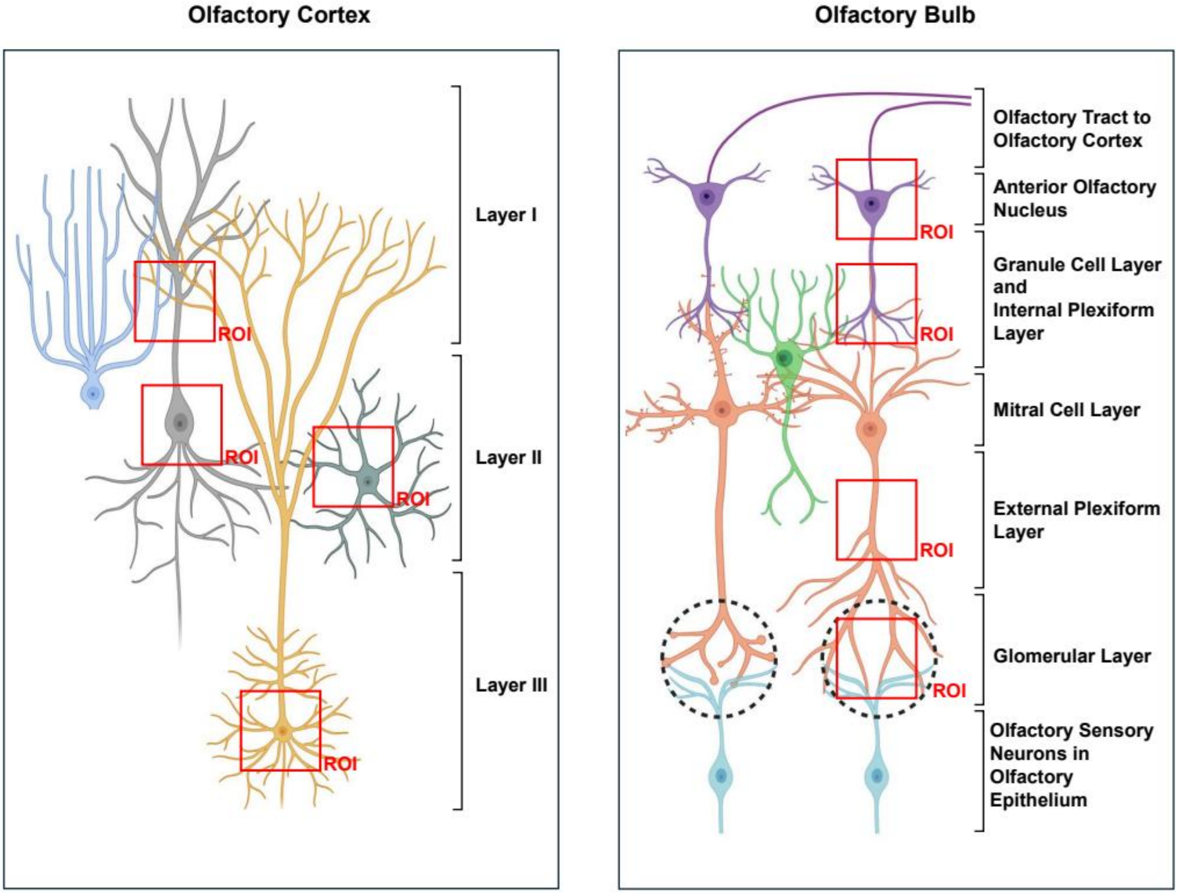

Supplementary Figure 2. Representative schematic of ROI placement within the OC and OB.

Representative schematic showing ROI placement within the OC (left) and OB (right) across CN, MCI, and AD cases. In the OC, ROIs were selected from Layer I (1 ROI), Layer II (2 ROIs), and Layer III (1 ROI). In the OB, ROIs were selected from the anterior olfactory nucleus (AON) (1 ROI), granule cell layer (GCL) (1 ROI), external plexiform layer (EPL) (1 ROI), and glomerular layer (GL) (1 ROI).

Marker	Region	Age (Spearman)		Sex (Mann-Whitney U)
		ρ	p value	p value
Aβ	OC	0.3160	0.1519	0.2703
	OB	0.2226	0.2959	0.4776
pTau	OC	0.2445	0.2727	0.8470
	OB	0.2841	0.1785	0.8428
Iba1	OC	0.3841	0.0776	0.7969
	OB	0.2701	0.2017	0.5512
GFAP	OC	0.2479	0.2659	0.6994
	OB	0.2016	0.3448	0.4776
ApoE	OC	0.1946	0.3855	0.4385
	OB	0.2221	0.2969	0.1782

Supplementary Table 1. Associations of age and sex with percentage-positive area by marker and region.

Spearman's rank correlations (ρ) were used to assess associations between age at death and per-case mean percentage-positive area for each marker in the olfactory cortex (OC) and olfactory bulb (OB). Sex-related differences were evaluated using two-sided Mann-Whitney U tests. Analyses were performed on the full available dataset for each marker/region; p values are unadjusted.

Abbreviations: OB, olfactory bulb; OC, olfactory cortex.

Marker	Region	p value (original)	R^2 value (original)	p value (excluded)	R^2 value (excluded)
Aβ	OC	<0.0001	0.8296	<0.0001	0.8128
	OB	0.0004	0.5214	0.0003	0.5785
pTau	OC	<0.0001	0.9541	<0.0001	0.9628
	OB	<0.0001	0.8308	<0.0001	0.8169
Iba1	OC	<0.0001	0.6994	<0.0001	0.6759
	OB	<0.0001	0.6797	<0.0001	0.6922
GFAP	OC	<0.0001	0.7387	<0.0001	0.7166
	OB	<0.0001	0.7499	<0.0001	0.7605
ApoE	OC	0.0029	0.4594	0.0019	0.5206
	OB	0.0074	0.3733	0.0094	0.3884

Supplementary Table 2. Sensitivity analysis excluding age-extreme cases (oldest AD and youngest CN): mean percentage-positive area by marker and region.

Sensitivity analysis assessing robustness of group effects to age-extreme sampling. For each marker, one-way ANOVA was performed separately in the olfactory cortex (OC) and olfactory bulb (OB) using per-case mean percentage-positive area. “Original” reports results from the full dataset; “Excluded” reports results after removing the oldest AD case and the youngest cognitively normal (CN) case within each region. The table reports ANOVA p values and effect size (R^2).

Abbreviations: OB, olfactory bulb; OC, olfactory cortex.
